# Lord Oxygen

**Published:** 1860-12

**Authors:** Geo. Watt


### LORD OXYGEN.

From an introductory to a course of Lectures on Chemistry.

BY GEO. WATT.

Man is, to a great extent, a creature of habit. He dislikes sudden changes. The schoolboy lingering on the bank, shrinking from the sudden plunge into the cold stream, demonstrates this truth to himself, while the pallid cheek and compressed lips of the commander about to order a charge, demonstrate the same truth to others. A disposition to hesitate precedes all great undertakings; and this is the great cause of that procrastination, known as "the thief of time," which, manifesting itself mainly in reference to repentance and matrimony, peoples this world with old bachelors, and the next with reprobates. About to engage in a great undertaking, shall we not be allowed to indulge in the luxuries of this universal law of laziness? About to embark for a four months' voyage, on the deep sea of an abstruse science, with imperfect charts, and inexperiened sailors, is it any wonder we incline to linger in port, and while away an hour, in view of the long toil before us? Let us, then, prove ourselves members of the great human family of procrastinators, by spending this hour, with less of toil and mental labor than will fall to our lot in the hours which are to succeed it.

But man is also a creature of instinct. He can be taught to know and do many things; but some things he will, and from his very nature, must know and do without any teaching, and even in spite of instruction to the contrary. He eats when he is hungry, drinks when he is thirsty, seeks for warmth when he is cold, and for a cool place when he is hot, not because his reason teaches him to do so, but simply in obedience to the instincts of his nature. Another, and one of his strongest instincts, impels him to worship that which he regards as worshipful. This instinct affords him no guide at all as to what he should worship. To do this, is the province of reason and revelation. And while these infallibly point out the great Creator of all, as the only fit object of worship, in the highest sense of the term, yet there is a sense in which "gods many, and lords many," may and should be worshiped, (or honored) with true devotion. But even here, reason and revelation must be his guide, or he will sink to the folly and crime of gross idolatry.

All worship, even in this subordinate sense, should be consistent. Consistent idolatry is far less odious than that which is inconsistent. We might pardon the astronomer for his worship of the stars; but we would be disgusted with his homage to a golden calf. Every man should be engaged in a good cause, and should admire, honor, or worship it accordingly.

Being now about to engage in the study of an important science, let us ask ourselves if the cause is not a good one. And if good, shall we not honor ourselves by honoring it? As the youth seeks the society of her whom he adores, so let us manifest our love for this science, by seeking an intimate acquaintance with its truths.

But if you doubt the propriety of rendering homage to this science, and fear that in doing reverence to it, in all its ramifications, we may be regarded as "too superstitious," let us turn our attention to a single element which it reveals to us, and inquire to what extent, if at all, it is entitled to our admiration.

What then, is the character of oxygen? To what extent is it entitled to our consideration? How much respect do we owe it? What reverence must we pay it? Shall we worship it?

Man naturally admires and reverences that which is mysterious. And this element is invisible. It is *seen* only in its works, which are many and wonderful. It forms the ocean and the land. It presides over the atmosphere, and governs the changes which take place on the earth's surface. The other elements are its servants, and are forced to aid in its manifold labors. With one it forms the rain-drop and the dew, with another, the balmy atmosphere, with a third, the the flinty rock, and with a fourth, the miry clay With one it fans the genial fire, and with another extinguishes the destroying conflagration. These are but parts of its ways—ever busy, but still invisible.

Nor is this all of its mystery. Where is it? Nay, where is it not? Is it omnipresent? It extends high above the mountain crag, and far below the ocean's bottom. It accompanies, or rather carries, the eagle in his most daring flight, and follows the miner down deep into the bowels of the earth, and is there even before him. It rides on the swift wings of the wind, and rejoices in the storm-cloud; for both are creatures of its construction. It is in the middle of the mountain, in the solid rock, and in all things that live. Man can not define its boundaries. To him it is, practically, omnipresent.

But think of its power. In the morning of creation it combined with the lightest and least tangible of all its comrades, and formed the fountains, rivers, seas, and oceans. In like manner, by other combinations, it made the solid earth. It seized a bright metal and turned it to lime. It laid hold on the diamond and changed it to a gas. Forcing these to unite, it formed the solid marble. It raised itself on the wings of the wind and became the vital principle of the atmosphere. And having formed the seas, the dry land, and the air, it presided over and took part in the formation of plants and animals to beautify and people the earth. And ever since it has been busy, and still it is powerful. It organizes the forces of its kindred elements, and works wonders at which man stands aghast. We behold the majestic steamer, stemming the river's current or riding on the angry waves, defying the wrath of the mighty deep, mocking at wind and tide, and reaching in safety its destined port, and all through the agency of this grand and glorious element. The ocean having spread itself out as a barrier to the progress of man, this hero-element comes to the rescue. Taking his favorite partner, the delicate hydrogen, he goes into the very heart of the mighty engine, and together, they form its life-blood. He calls for his servant carbon, and fans him into a flame, imparting warmth, and life, and motion, to the mighty machine of his own building; and the ocean is subdued, the winds are overcome, places far distant are brought nigh together, man becomes ubiquitous, and all are neighbors. With the same mighty machine he drags the lightning train across the land, over iron bars of his own forging, laughing at distance and mocking at time. His lightning train over his iron track! Nay, it is thus he travels, at leisure. When pressed for time, he calls down the lightnings of heaven, and sends them forth across land and sea, on the wiry track which he has prepared for their guidance. May we not well exclaim, O Oxygen, great and marvelous are thy works!

But our hero element is powerful to destroy, as well as to build up. Few, if any, elements have hardened themselves against him, and have prospered. The strength of iron is as nothing with him, and weapons of steel he grinds to powder. Gold becomes as fine dust before him, and silver as the blackness of darkness. He calls for the aid of his servant, nitrogen, and the solid marble melts like snow. Helped by another servant, the flinty rock becomes grass and stubble before him. The leaves wither at his blighting touch, and nature is dissolved by his blasting energies. Let us think, for a moment, of the ruin he has wrought, since creation's morn. The flowers, the trees, the birds and the beasts, of ancient days, have all been swept away by the hand of this ruthless destroyer. He has swept over the earth, and nations have withered, by the blast of his breath. Great Babylon is fallen—is fallen, and by his mighty hand. Where are the cities of the old world? He has burned them to ashes, or crumbled them to dust; and, over their ruins, he raises the shout of triumph, and rushes forward to new conquests. When the whole world had rebelled, he destroyed it by a flood—all its inhabitants save one righteous family, which he carried in his bosom, and wafted, by the genial gales of his balmy breath, to a place of safety. He has broken the ships of Tarshish with his east wind. He has overwhelmed fleets and navies in the mighty deep. Great is the ruin he has wrought, and still he is unsatiated. He forges the thunderbolts of war, and gives to them their destructive energy. He manufactures a cooling salt, which, in the hand of two of his servants, becomes a demon of destruction, hurling forth "firebrands, arrows and death," and imitating the thunders of heaven. Nothing material escapes his destroying hand. He devours the widow's bread, and wastes the fruits of the earth, to create the demon alcohol, that the world may be filled with crime, and men may be changed to devils. He is the merciless executor of that sentence, "Dust thou art and unto dust shalt thou return."

And then just think of his petty annoyances. While he blows the smith's fire, he consumes his coals, and wastes his iron. The surgeon rejoices in his shining blade, and the dentist delights in the lustre of his forceps, but he watches for their negligence and covers them with rust. He withers the leaves of the lady's arbor, and sours the milk in the dairymaid's pans. No annoyance is so petty that he will not stoop to it. He addles the egg of the patient bird, and molds the food of the busy ant. He breathes into the pantry and the bread becomes stale, the butter rancid, the meal musty and the meat tainted. He rots the farmer's fruits, his farm houses and fences, and turns the housewife's jellies and jams to vinegar. In short, he is prying, petulant and impertinant.

Shall we reverence such an object as this? Shall we even respect it? Nay, shall we not ignore or despise the science that discovers it and reveals its attributes? But why should we not reverence it? Why not even worship it? While sages sacrifice to devils, and savages worship the storm-king, shall we fail to worship oxygen? What if it is the great destroyer of our race? Should we not try to propitiate that which will one day crumble us to dust?

But let us turn from this and think of his goodness. Why, he blesses us every hour—gives us a new blessing with every breath. But how shall we specify his acts of kindness when we owe him our life, and are each moment dependent on him for its continuance. In the earliest moments of our helpless infancy, he breathed into our nostrils the breath of life, and has breathed new life ever since. He allays our hunger, and quenches our thirst, with food and drink adapted to our appetites and desires. He not only surrounds us with good things, but he is so jealous of our welfare that he rushes into and explores every avenue of our bodies lest there may be something there to harm us. He ransacks our entire systems —veins, arteries, capillaries, and cavities—and comes out loaded with poison twenty times a minute. He makes every pore of the skin an outlet for the purification of our bodies. He kindles a fire to warm us when we are cold, and fans us with the cooling breeze when we are hot. He builds us houses, like palaces, to dwell in, for our comfort and protection. It would seem as if he were determined that man should live always—not only live but that he should riot in all the imaginary luxuries of a fabled dream.

Not content that man should live—that he should breathe the balmy air of a life-giving atmosphere, that he should slake his thirst from the sparkling spring, and eat of that which is good, and delight his soul in fatness—our favorite element overwhelms him with delights, of which he could not even dream, were they not constantly showered upon him as happifying realities. How he blesses our sight! He clothes the earth with its carpet of green, and covers the forests with the same gay colors.

"Gay green! thou smiling nature's universal robe"—

And our hero is the dyer that colors that robe. He paints the flowers with their varied hues, he screens the sunset with its crimson vail, and curtains the heavens with the fleecy cloud. And think of his beauty as he glistens in the dew-drops, and sparkles in the rain, or when he spreads the canvas of the storm-cloud, and illuminates his face with the day-god's pencilings, in the hues of the bow of promise. He moistens the eye of the maiden till it glistens with love. He fans her check, till it rivals the rose, and bleaches her brow to the lily's hue. And even when he appears to frown—when the face of nature is wrapt in decay, he makes her beautiful, even in death. When the forest leaves fade—as fade they must, for they are mortal—he beautifies their death-robes, with his varied tints, till the eye is entranced with the gorgeous colors, and even the south wind accepts them as a substitute, and no longer

"Searches for the flowers
Whose fragrance late he bore,
And sighs to find them in the wood,
And by the stream no more."

But not only does he delight the eye, but he charms us with the melody of nature. He teaches the bee to hum, the bird to warble, and the child to laugh. He gives voice to the singer, and tones to the lute and organ. In short, he blesses us through all our senses.

Nor is he at all capricious in the bestowal of his favors. In springtime he moistens the earth with showers, fans it with gentle breezes and bedecks it with flowers. He makes the grass for the cattle, and the tender herb for the service of man. In summer he comes riding on the south wind, and warms and fertilizes the earth. He loads the trees with fruit, and causes the grain to grow, for the sustenance of man and beast. In autumn, he blesses us with the fruits of the earth, and satisfies us with the increase of the ground. The harvest of the earth is ripened by his breath, and the husbandman rejoices in the abundance of his blessings. In winter, he converts even the fierce north wind into a blessing. He scatters hoarfrost like ashes, or spreads it, in silvery beauty, over the window of the sleeper. He covers the earth with snow, and makes us rejoice in the tinkle of the sleighbell. At all times and seasons he is the same kind benefactor.

He disburses his favors not only with a liberal hand, but without partiality. His goodness reaches to all men. Every department of life is blessed by his presence, and aided by his energies. He rewards the labors of the farmer with abundant crops. He brings merchandise from afar, over sea and land, to enrich the trader. He blows the fire for the smith, and turns the mill-wheel and the spindle. He refines the ores of the metallurgist, and melts the founder's metals. To the physician and the dentist he is indispensable. He prepares the medicines of the one, and purifies the metals used by the other. No department of life is beneath his notice. He toils in the kitchen as a faithful servant preparing and baking the bread, roasting the meat, browning the coffee, and drawing the tea. He covers our tables with luxuries, and respects the appetites of the most whimsical. He fills the sails of the mariner, and wafts him to his destined port. He floats the schoolboy's tiny boat, and carries aloft his paper kite He furnishes the painter his colors, and the sculptor his clay and marble. He carries the bird in its rapid flight, and goes deep down into the dark ocean, to bless the great whale and the little fish. He stops at nothing in his errands of mercy. Floods can not drown him, and fire is but a plaything, of his own creation. Hight and depth are alike to him, and distance is only his delight. If not revered for his wonderful works, should he not be praised for his goodness?

Here, then, is presented to our consideration an agent, invisible, diffused, mysterious, powerful for good and powerful for evil, that goes about doing good, and, at the same time, seeking what he may destroy, that kills and makes alive, that wounds and heals. whose existence antedates the life of man, which is irdestructible as well as unchangeable. However we may estimate it, one thing is certain, that if it had been revealed by our science, in ancient days, the great Jupiter would have been deposed, to at least secondary rank. Had it been known at Athens, the apostle would not have found an altar "to the unknown God," but to the god, Oxygen. And when we reflect on the characters of the gods they worshiped, we are led to pity their ignorance of this wonderful agent, which seems so much more worthy of the homage of their philosophers. But while we pity, let us inquire if our worship of this would be found more exalted and purifying than their worship of beasts and reptiles, or wood and stone.

What, then, is Oxygen? Certainly it is a wonderful thing; for a wonderful God created it. And because it is a creature, and not a creator, it stands on a level with its kindred elements—on a level with birds, and beasts, and reptiles, and is no more wonderful than they. In the light of our science, every thing that God has made is wonderful, and, to us, incomprehensible. Everything is worthy of our admiration; and it is highly proper that the science we are about to study, which has a more extensive range, and reveals more of the mysteries of the material universe, than any other you are called upon to investigate, should teach us to "look through nature up to nature's God," to see the Creator in all his works, to look upon all the elements as but so many passive instruments in his hand. Till we are able to do this, we have failed to learn the great lesson taught by our science.

But, gentlemen, do you expect to master the science of chemistry, in the few short months we are to spend together? As well might the traveler expect to traverse the entire globe in a single day, and return at nightfall to sleep in his cottage. No man on earth will ever get through with the study of this science. But shall we despair on this account? May it not be

"That one of the joys of our heaven shall be"

a fuller and clearer knowledge of the chemical properties of God's universe, than it is possible for us to have here? But you may expect to obtain, by attention and study, such knowledge of it as will make all future study of it a pleasure, and a source of satisfaction.

Are you discouraged and ready to turn back, on finding that you make so little progress in this science? You have the same cause for discouragement elsewhere; for you have not, and never will have a perfect knowledge of any science; nor would you be happy if you had. Here we know only in part; but there—we shall know, even as we are known.

